# Black Sorghum Phenolic Extract Regulates Expression of Genes Associated with Oxidative Stress and Inflammation in Human Endothelial Cells

**DOI:** 10.3390/molecules24183321

**Published:** 2019-09-12

**Authors:** Nidhish Francis, Shiwangini Rao, Christopher Blanchard, Abishek Santhakumar

**Affiliations:** 1 School of Animal and Veterinary Sciences, Faculty of Science, Charles Sturt University, Wagga, NSW 2650, Australia; nfrancis@csu.edu.au; 2 Australian Research Council (ARC) Industrial Transformation Training Centre for Functional Grains, Graham Centre for Agricultural Innovation, Charles Sturt University, Wagga, NSW 2650, Australia; srao@csu.edu.au (S.R.); cblanchard@csu.edu.au (C.B.); 3 School of Biomedical Sciences, Faculty of Science, Charles Sturt University, Wagga, NSW 2650, Australia

**Keywords:** black sorghum, genes, inflammation, oxidative stress, polyphenols

## Abstract

Oxidative stress is one of the primary factors leading to endothelial dysfunction, a major underlying cause of vascular disorders. This study aims to understand the key signalling pathways regulated by sorghum (Shawaya short black 1 variety; characterised to be very high in its antioxidant activity) under oxidative stress in endothelial cells. Human umbilical vein endothelial cells (HUVECs) were pre-treated with non-cytotoxic concentrations of phenolic-rich black sorghum extract (BSE) prior to induction of oxidative stress using hydrogen peroxide (H_2_O_2_). Treatment with BSE upregulated the expression of heme oxygenase 1 (HO1) and endothelial nitric oxide synthase (eNOS) and downregulated the levels of NADPH oxidase 4 (NOX4). BSE treatment significantly reduced the expression of pro-inflammatory mediators such as monocyte chemoattractant protein 1 (MCP1) and intracellular adhesion molecule 1 (ICAM1). Results from this study suggest that phenolic-rich BSE may reduce oxidative stress by regulating pro- and antioxidant signalling pathways and the expression of inflammatory mediators linked to endothelial dysfunction under oxidative stress.

## 1. Introduction

Endothelial cells lining the blood vessels form part of an active endocrine, paracrine and autocrine organ that produces and secretes multiple bioactive signalling molecules necessary for vascular homeostasis [1]. Endothelial dysfunction is considered to be the principal cause of many cardiovascular diseases and is the initial step in the pathogenesis of atherosclerosis [2]. Oxidative stress, the perturbation of oxidation-reduction balance inside the cell leading to excess production of reactive oxygen species (ROS) such as superoxide (O_2_^−^), hydroxyl radical (.OH), hydroxyl ion (OH^−^) and hydrogen peroxide (H_2_O_2_) contributes to endothelial dysfunction. The excess ROS production potentiates the activation of various inflammatory signalling pathways, ultimately resulting in cell death [3].

Endothelial cells under oxidative stress regulate the activation of many signalling pathways. Nuclear factor-E2-related factor 2 (Nrf2), a transcription factor protein, is one such signalling molecule that is activated during oxidative stress [4]. Nrf2 is normally bound to kelch-like ECH-associated protein 1 (Keap1) in the cytoplasm under normal conditions for ubiquitination and degradation. Under oxidative stress, Nrf2, released from Keap1, translocates into the nucleus and induces the expression of antioxidant genes such as heme oxygenase 1 (HO1) and NADPH quinone oxidoreductase 1 (NQO1).

The nicotinamide adenine dinucleotide phosphate (NADPH) oxidase system comprising NOX1, NOX2, NOX4 and NOX5 serves as a major source of ROS production [5]. In particular, NOX4 is reported to be associated with the formation of early atherosclerotic plaque [6]. Another mechanism for the regulation of endothelial function is the production of endothelium-derived nitric oxide (NO), mediated by endothelial NO synthase (eNOS) [7]. Adequate amounts of NO in the vasculature ensure tonicity. Studies have demonstrated that under oxidative stress, the bioavailability of NO is significantly lowered, partly due to the lowered levels of eNOS expression.

An increase in the inflammatory state of the endothelial cells due to oxidative stress elevates the expression of other inflammatory mediators such as cell adhesion molecules, chemokines and ectonucleotidases. Studies have demonstrated that injured endothelial cells have elevated levels of intracellular adhesion molecule 1 (ICAM1) and vascular cell adhesion molecule 1 (VCAM1) [8,9]. Monocyte chemoattractant protein 1 (MCP1) is a chemokine produced by human endothelial cells, mononuclear phagocytes and fibroblasts in response to cell damage, thereby stimulating the migration of monocytes to the site of injury. It has been shown that MCP1 is one of the key chemokines to be tightly regulated during vascular inflammation and other metabolic disruptions [10]. Ectonucleotidases are known to be regulators of purinergic signalling, important for inflammation and immunity functions [11,12]. A loss in the endothelial cell integrity during inflammation results in the leakage of ATP extracellularly that is subsequently converted to adenosine monophosphate (AMP), mediated by the ectonucleotidase, CD39. Another ectonucleotidase, namely CD73, later catalyses the conversion of the newly formed AMP to adenosine, which results in anti-inflammatory and immunomodulatory effects [11,12].

There has been an increasing interest in functional foods due to the added health benefits they may provide and recent research has focused on identifying the mechanisms associated with their disease preventive or therapeutic potential. One such example for a functional food is sorghum whole grain. Several varieties of sorghum exist, namely black, red, brown and white and are classified based on the pigmentation of the pericarp [13]. Currently, sorghum is primarily used as an animal feed for cattle, pigs and poultry in Australia. Recent studies have demonstrated that sorghum has anti-oxidant, anti-inflammatory, anti-microbial and anti-cancerous properties [14], thereby adding value to sorghum grains and increasing human consumption. The antioxidant and anti-inflammatory activity observed in the pigmented varieties of sorghum is primarily attributed to its phenolic content. Several polyphenols such as flavonoids, hydroxybenzoic acids and hydroxycinnamic acids have been identified in sorghum and the concentration of these compounds vary according to the genotypes and production environments [14,15]. The anthocyanin derivative, 3-deoxyanthocyanidin (3DXA), is a unique bioactive compound found in sorghum and extensively studied for its high antioxidant activity [16]. In addition to 3DXA, sorghum has many other polyphenols that may also contribute to its antioxidant activity such as catechins and their derivatives [14]. Although recent research has attempted to gain an understanding of the antioxidant and anti-inflammatory properties of sorghum-derived polyphenols [14,17], underlying molecular mechanisms that contribute to these properties remain unclear. Therefore, the aim of the present study is to investigate the gene expression profiles for potential antioxidant and anti-inflammatory signalling pathways regulated by polyphenols derived from black sorghum in oxidative stress-induced human endothelial cells.

## 2. Results

### 2.1. Cytotoxicity of Phenolic-Rich Black Sorghum Extract (BSE) on HUVECs

**Figure 1:**
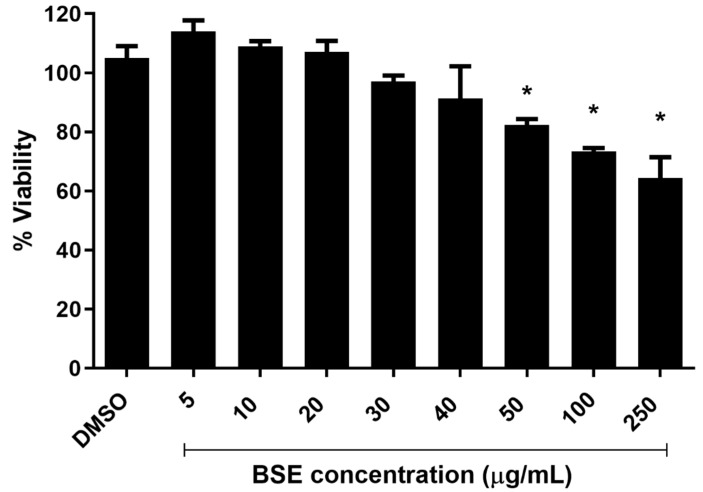
Cytotoxicity of phenolic-rich BSE on HUVECs. HUVECs were treated with BSE at various concentrations for 2 h and followed by resazurin red cytotoxicity assay. *n* = 3. Level of significance indicated as * *p* < 0.05, one-way ANOVA with Tukey’s multiple comparison post hoc test. Data is presented as mean ± SEM. DMSO—dimethyl sulfoxide, HUVEC—human umbilical vein endothelial cells, BSE—black sorghum extract.

A significant (*p* < 0.05) reduction in the viability of cells was observed when supplemented with BSE at concentrations of 50, 100 and 250 µg/mL when compared to the control group (0.05% dimethyl sulfoxide (DMSO)) for 2 h using the resazurin red cytotoxicity assay. It has already been established that the polyphenols such as flavonols, flavonones, catechin and their derivatives are bioavailable in the human body for a maximum of 2–3 h [18]. Taking this into consideration, our experimental design included treatment of human umbilical vein endothelial cells (HUVECs) with the phenolic-rich extract for 2 h as previously reported [19]. No significant difference in the viability of HUVECs was observed for treatment groups supplemented with 5, 10, 20, 30 and 40 µg/mL of phenolic-rich BSE (Figure 1). BSE at concentrations of 5, 20 and 40 µg/mL was chosen for further gene expression studies.

### 2.2. Phenolic-Rich BSE Regulates the Expression of Oxidative Stress-Induced Antioxidant Pathway-Related Genes

**Figure 2:**
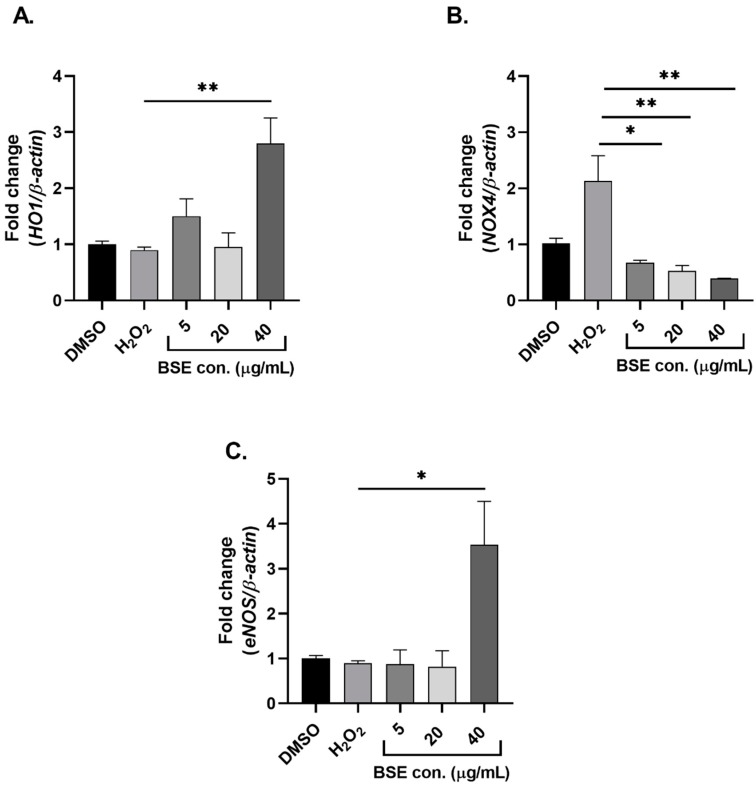
Changes in the expression profile of antioxidant genes [*HO1* (**A**), *NOX4* (**B**) and *eNOS* (**C**)] with BSE pre-treatment on oxidative stress-induced HUVECs. *n* = 3. Level of significance indicated as * *p* < 0.05, ** *p* < 0.01, one-way ANOVA with Tukey’s multiple comparison post hoc test. Data is presented as mean ± SEM. DMSO—Dimethyl sulfoxide, HUVEC—human umbilical vein endothelial cells, BSE—black sorghum extract.

The candidate antioxidant-related genes assessed to investigate the effect of phenolic-rich BSE on endothelial cells were *Nrf2*, *NQO1*, *HO1*, *eNOS* and *NOX4*. Induction of oxidative stress post BSE supplementation at 40 µg/mL significantly (*p* < 0.05) upregulated the expression of *HO1* and *eNOS* when compared to the positive control and the group treated with H_2_O_2_ only (Figure 2A,C). A significant (*p* < 0.01) dose-dependent downregulation in the levels of *NOX4* post BSE supplementation was observed in comparison to the control (Figure 2B). However, BSE supplementation did not regulate the expression of *Nrf2* and *NQO1* at any concentrations tested (data not shown).

### 2.3. Phenolic-Rich BSE Regulates the Expression of Oxidative Stress-Induced Inflammatory Pathway Genes

**Figure 3:**
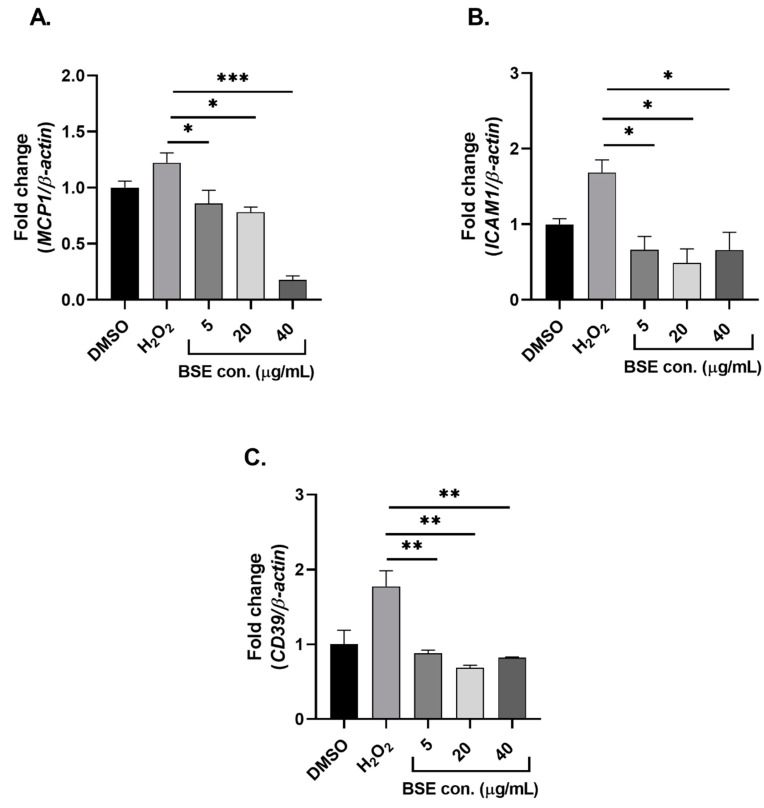
Changes in the gene expression profile of inflammatory mediators [*MCP1* (**A**), *ICAM1* (**B**), and *CD39* (**C**)] with BSE pre-treatment on oxidative stress-induced HUVECs. *n* = 3. Level of significance indicated as * *p* < 0.05, ** *p* < 0.01, *** *p* < 0.001 one-way ANOVA with Tukey’s multiple comparison post hoc test. Data is presented as mean ± SEM. DMSO—Dimethyl sulfoxide, HUVEC—human umbilical vein endothelial cells, BSE—black sorghum extract.

The expression of *ICAM1*, *MCP1*, *CD39* and *CD73* were determined post BSE supplementation and induction of oxidative stress. Supplementation of phenolic-rich BSE at 5, 20 and 40 µg/mL significantly (*p* < 0.05) downregulated the expression of *ICAM1* when compared to the control (Figure 3A). Under an oxidative stress-induced environment, a significant (*p* < 0.05) downregulation in the levels of *MCP1* and *CD39* was observed in all phenolic-rich BSE-supplemented groups (Figure 3B,C). Supplementation of phenolic-rich BSE did not alter the expression of *CD73* in HUVEC cells under oxidative stress (data not shown).

## 3. Discussion

Endothelial dysfunction, one of the key underlying causes of cardiovascular diseases, is strongly initiated by oxidative stress, which in turn induces the production of pro-inflammatory cytokines, chemokines and other immunomodulatory signalling molecules. Findings from this study demonstrate that phenolic-rich BSE regulates the expression of key antioxidant, anti-inflammatory and immunomodulatory genes. Supplementation of BSE significantly upregulated the expression of HO1 and eNOS and downregulated the expression of NOX4, MCP1, ICAM1 and CD39. The major phenolic compounds identified in the black sorghum phenolic-rich extracts used in this study are catechins and their derivatives (Table S2) [14]. The highest level of phenolic compound characterised in the BSE was a catechin derivative (2.11 ± 0.47 mg 100 mg^−1^ Gallic Acid Equivalents [GAE]) followed by catechins (1.97 ± 0.36 mg 100 mg^−1^ GAE) and pentahydroxyflavanone-(3->4)-catechin-7-*O*-glucoside (1.80 ± 0.28 mg 100 mg^−1^ GAE). The highest antioxidant activity analysed through online-ABTS system was observed for pentahydroxyflavanone-(3->4)-catechin-7-*O*-glucoside (2.62 ± 0.36 mg 100 mg^−1^ TE) followed by 1-*O*-caffeoylglycerol-O-glucoside (1.74 ± 0.33 mg 100 mg^−1^ TE) and catechins (1.54 ± 0.25 mg 100 mg^−1^ TE). It is to be noted that the compounds such as catechins and pentahydroxyflavanone-(3->4)-catechin-7-*O*-glucoside found in greater quantities in the extract also exhibited relatively higher antioxidant activity suggesting that the antioxidant effects observed with BSE treatment is primarily contributed by these phenolic compounds.

Several factors regulate the expression of the Nrf2-Keap1 signalling pathway under oxidative stress. Numerous phenolic compounds in resveratrol, curcumin, genistein and catechins have been demonstrated to activate the Nrf2 pathway [20]. A study on the antioxidant activity of acai (*Eutrepe oleracea Mart.*) seed extract demonstrated an upregulation of Nrf2 antioxidant signalling levels to prevent oxidative stress in human endothelial cells [21]. In this study, phenolic-rich BSE did not induce the expression of Nrf2 but upregulated the expression of HO1, typically a Nrf2-regulated gene. At 40 µg/mL of phenolic-rich BSE supplementation, the expression of HO1 was significantly upregulated in HUVECs post H_2_O_2_ treatment when compared to the positive control. This is indicative of the cytoprotective effect of phenolic-rich BSE on the endothelial cells under oxidative stress. This result is consistent with a previously published study where the treatment of HUVEC cells with salidroside, one of the major constituents of *Rhodiola rosea*, demonstrated a dose-dependent significant upregulation of HO1 expression under H_2_O_2_-induced oxidative stress conditions [22]. Interestingly, a significant change in the expression of HO1 but not Nrf2 in the current study is suggestive of alternate pathways that may regulate HO1 expression completely independent of Nrf2 activation. An in vivo model of skeletal muscle atrophy demonstrated a significant upregulation of HO1 levels in Nrf2 knockout mice suggesting that HO1 regulation can occur independent of Nrf2 [23]. Furthermore, this study also reported that HO1 is regulated by forkhead box protein O1, another major regulator of oxidative stress [23]. In another study on keratinocytes, it was demonstrated that HO1 induction during keratinocyte differentiation occurred through Nrf2-independent pathways [24]. These studies thereby suggest that HO1 expression could be regulated independent of Nrf2. Among the various isoforms of NOXs (NOX1, 2, 4 and 5) identified, human endothelial cells primarily express NOX4 [25], which was the rationale for investigating changes in the expression of NOX4 in our current study. To date, there are no published reports investigating the effect of sorghum-derived polyphenols on NOX4 expression. Other plant extracts such as polysaccharides extracted from Rhizoma Dioscoreae Nipponicae significantly inhibited the overexpression of NOX4 and p22hox (a critical component of the NADPH oxidase system) induced by H_2_O_2_ [26]. Similarly, extracts from *Piper sarmentosum* and Chinese herb danshen (*Salvia miltiorrhiza* L.) have been shown to inhibit the expression of NOX4 in oxidative stress-induced human endothelial cells [27,28]. We observed a significant upregulation of NOX4 expression under oxidative stress and pre-treatment with phenolic-rich BSE significantly decreased expression of NOX4. This suggests that BSE may act as a ROS scavenger eliminating the free radicals generated from many signalling pathways including the NADPH oxidase system, thereby preventing endothelial cells from significant oxidative stress damage.

Endothelial NO synthase (eNOS), which is primarily responsible for the production of nitric oxide (NO), provides a physiologically protective role in the vasculature. In this study, we observed that the expression levels of eNOS were upregulated when supplemented at a higher concentration of phenolic-rich BSE demonstrating the cytoprotective effect of BSE on human endothelial cells under oxidative stress. Consistent with results from our study, flavonoids from artichoke (*Cyanara scolymus* L.) have been shown to upregulate the expression of eNOS in human endothelial cells [29]. Similarly, extracts from the leaves of *Ribes nigrum* L. stimulated the expression of eNOS and thereby increased the production of NO in cultured endothelial cells [30]. It is to be noted that upregulation of eNOS expression is not always cytoprotective and at times can be harmful. An uncoupling of eNOS occurs at certain pathophysiological conditions, leading to the generation of superoxide ion rather than normal production of NO. These superoxide ions generated by the uncoupled eNOS enhances the existing oxidative stress, resulting in the deterioration of cardiovascular disease states [31].

Several studies have demonstrated changes in the levels of cell adhesion molecules such as ICAM1, VCAM1 and selectins in response to oxidative stress in endothelial cells which result in the progression of endothelial dysfunction [8,28]. Results from this study showed that the increased expression of ICAM1 under oxidative stress is inhibited by pre-treatment with phenolic-rich BSE. It is interesting to note that there was a significant downregulation of ICAM1 levels at very low concentrations of BSE supplementation (5 µg/mL) strongly reflecting the potent anti-inflammatory properties of phenolic compounds isolated from black sorghum. A study investigating the effect of coloured rice derived phenolic-rich extracts on oxidative stress demonstrated a significant reduction in the levels of ICAM1 [19]. Similarly, studies on other plant-derived extracts have also demonstrated a significant downregulation of ICAM1 expression in oxidative stress-induced human endothelial cells [26,28]. These investigations of the changes in ICAM1 expression are aligned with the findings from this study.

One of the well-known chemokines known to exacerbate inflammation and promote the progression of endothelial dysfunction is MCP1. Results from this study demonstrated a dose-dependent downregulation in the expression of MCP1 upon pre-treatment with phenolic-rich BSE in oxidative stress-induced endothelial cells. There was more than an 80% reduction in the levels of MCP1, when supplemented with 40 µg/mL of BSE. Several reports demonstrate the action of MCP1 in being a strong inflammatory mediator and most of these studies focus on the production of MCP1 from monocytes and macrophages [32,33]. To date, there are very few studies that have investigated the changes in the expression of MCP1 produced specifically by endothelial cells. A study on the toxicological effect of silica nanoparticles on human endothelial cells demonstrated a significant upregulation of MCP1 along with other pro-inflammatory molecules such as IL-6, TNF-α, ICAM1 and VCAM1 [34]. Recent reports on the ectonuleotidases, CD39 and CD73, highlighted these genes as potential candidates to be analysed in our current study. Studies have demonstrated that both CD39 and CD73 possess immunomodulatory function by dampening the immune responses upon their activation [12]. In this study, pre-treatment with BSE prevented the upregulation of CD39 in oxidative stress-induced HUVECs suggesting that BSE supplementation reduced the overall oxidative stress state and production of inflammatory mediators in HUVECs.

In summary, this study demonstrated that phenolic compounds derived from a black sorghum variety significantly altered the expression of key antioxidant- and inflammation-linked genes (*HO1*, *eNOS*, *NOX4*, *MCP1*, *ICAM1* and *CD39*) in oxidative stress-induced human endothelial cells. It is very likely that catechins and their different isomers abundantly found in the BSE used in this study act synergistically, under oxidative stress, to elicit an antioxidant and anti-inflammatory effect. Future studies analysing the activity and expression of these identified candidate biomarkers at protein level and in vivo investigations determining the bioavailability of the phenolic-rich black sorghum extract will confirm and provide implications for its use in preventing oxidative stress-induced endothelial dysfunction.

## 4. Materials and Methods

### 4.1. Sorghum Samples and Phenolic Extraction

Sorghum (*Sorghum bicolor*) samples were obtained from glasshouse trials performed at Curtin University, Perth Australia. The black pericarp variety of sorghum (Shawaya short black 1) was selected for this study due its previously identified potent antioxidant activity [14]. Ferric reducing antioxidant power (FRAP) assays have demonstrated that extracts from the black pericarp variety exhibited high antioxidant activity (20.92 ± 2.69 mg/g TE). Similarly, results of 2,2-diphenyl-1-picrylhydrazyl (DPPH) antioxidant assays also demonstrated a high antioxidant activity for extracts from this variety (18.04 ± 3.53 mg/g TE) [14] compared to other varieties. Key findings are provided in the Supplementary Figure S1 and Table S1. Phenolic extraction was performed using methods described by Rao et al. [14].

### 4.2. Cells and Culture Conditions

Human umbilical vein endothelial cells (HUVEC) were purchased from Sigma-Aldrich (St Louis, MO, USA) and cultured in complete endothelial cell growth medium (Cell Applications Inc, San Diego, CA, USA). The cells were maintained in a 5% CO_2_ incubator at 37 °C. All assays for this study were undertaken using HUVECs between 9 and 11 passages.

### 4.3. Cytotoxicity Assay

The cytotoxicity of the black sorghum-derived phenolic rich extracts (BSE) was determined using the resazurin red cytotoxicity assay [19]. HUVECs were seeded onto 96-well plates at a density of 5 × 10^3^ cells per well in complete endothelial cell growth medium. After 24 h of incubation, the cells were treated with varying concentrations of BSE at 5, 10, 20, 30, 40, 50, 100 and 250 µg/mL for 2 h. After careful removal of BSE supplemented media, 200 µL of resazurin dye solution (14 mg/mL) was added to each well and incubated for 4 h at 37 °C in 5% CO_2_. Following this, 150 µL of the incubated dye was collected from each well without disturbing the monolayer of HUVECs at the bottom and transferred to new wells. Absorbance of the collected media was measured at 570 and 600 nm using a microplate reader (FLUOstar Omega, BMG Labtech, Offenburg, Germany). Endothelial cell growth medium supplemented with BSE served as the blank for each concentration analysed for cytotoxicity. A solution of 0.05% dimethyl sulfoxide (DMSO) was used as the negative control for all experiments performed in this study. The percentage viability of the cells was calculated using a formula published previously [19].

### 4.4. Experimental Design and Oxidative Stress Induction

HUVECs were seeded onto 6-well plates at a density of 3 × 10^5^ cells per well and maintained in the culture media and conditions described above. Twenty-four hours later, the old media was removed, and cells were incubated with growth media supplemented with 5, 20 and 40 µg/mL of BSE for 2 h. The BSE supplemented media was then removed, and cells were washed once with phosphate buffered saline (PBS). Wells supplemented with 0.05% DMSO served as the negative control. After washing with PBS, cells were supplemented with growth medium containing 200 µM of hydrogen peroxide (H_2_O_2_) and incubated for 1 h to induce oxidative stress. Wells not exposed to BSE supplementation but incubated with growth media containing 200 µM H_2_O_2_ served as the positive control. The experiment was performed in triplicate.

### 4.5. RNA Extraction and Reverse Transcription

Post BSE supplementation and H_2_O_2_ treatment, the cells were washed in ice-cold PBS. Following this, the total RNA was extracted using a Wizard SV RNA extraction kit (Promega, WI, USA), according to the manufacturer’s instructions. The purity of extracted RNA was assessed using a NanoDrop 2000c spectrophotometer (ThermoFisher Scientific, MA, USA) prior to reverse transcription. An aliquot of solution containing a total of 400 ng of RNA was used to generate cDNA using a GoScript Reverse Transcription kit (Promega, WI, USA).

### 4.6. Quantitative Polymerase Chain Reaction (qPCR)

Quantitative PCR was performed using SSoAdvanced Universal SYBR Green Supermix (Bio-Rad Laboratories, CA, USA) on a CFX96 Real-Time System (Bio-Rad, CA, USA). Primers for genes were designed using Primer3 software [35] and are listed in Table 1. Specificity of these primers was confirmed using the NCBI BLAST tool. All primers were tested for their amplification efficiency which ranged from 90 to 110% (Table S3). A single qPCR reaction mix consisted of 1 µL of 1:5 diluted cDNA, 5 µL of Supermix (Bio-rad, CA, USA) and 1 µM of forward and reverse primers. The thermal profile consisted of 95 °C for 3 min followed by 40 cycles of 95 °C for 10 s and 58 °C for 30 s. This was followed by a thermal melt profile from 65 to 95 °C. The dissociation curve generated by the thermal melt profile was investigated for the presence of a single PCR product before proceeding with analysis. The relative abundance of these genes was determined after normalisation with the housekeeping gene, β-actin. Q-gene software was used to calculate the mean normalised expression of genes of interest using the cycle threshold (Ct) values obtained for the target and housekeeping genes [36].

### 4.7. Statistical Analysis

All data were statistically analysed by one-way analysis of variance (ANOVA) coupled with Tukey’s multiple comparison post hoc test using GraphPad Prism software (version 8). Differences at values of *p* < 0.05 were determined to be statistically significant. Data are represented as mean ± SEM.

## Figures and Tables

**Table 1:** List of genes with their primer sequences used for qPCR.

Gene	Forward Primer	Reverse Primer
*Nrf2*	ATGACAATGAGGTTTCTTCGG	CAATGAAGACTGGGCTCTC
*NQO1*	ACATCACAGGTAAACTGAAGG	TCAGATGGCCTTCTTTATAAGC
*HO1*	AACTCCCTGGAGATGACTC	CTCAAAGAGCTGGATGTTGAG
*NOX4*	TATCCAGTCCTTCCGTTGG	CCAATTATCTTCTGTATCCCATCTG
*eNOS*	GTTACCAGCTAGCCAAAGTC	TCTGCTCATTCTCCAGGTG
*MCP1*	CCAGATGCAATCAATGCCC	TGGTCTTGAAGATCACAGCT
*ICAM1*	GATAGCCAACCAATGTGCT	TTCTGGAGTCCAGTACACG
*CD39*	TCAAATGTAGTGTGAAAGGCTC	TACACTCCTCAAAGGCTCTG
*CD73*	CATTCCTGAAGATCCAAGCA	AGGAGCCATCCAGATAGAC
*β-Actin*	GAAGATCAAGATCATTGCTCCTC	ATCCACATCTGCTGGAAGG

## Supplementary Materials

Supplementary Figure S1, Table S1 and Table S2 demonstrate the key findings from the study of Rao et al. [14] using the UHPLC online ABTS system, and DPPH and FRAP assays. Table S3 represents the amplification efficiency of primers used in this study.

## Abbreviations

3DXA	3-deoxyanthocyanidin
BSE	black sorghum extract
CD	cluster differentiation
eNOS	endothelial nitric oxide synthase
HO 1	heme oxygenase
HUVECs	human umbilical vein endothelial cells
H_2_O_2_	hydrogen peroxide
ICAM 1	intercellular adhesion molecule
MCP) 1	monocyte chemoattractant protein
NOX) 4	NADPH oxidase
NQO 1	NAD(P)H:quinone oxidoreductase
Nrf2	nuclear factor erythroid 2–related factor 2
