# Supplementary material for: Black Sorghum Phenolic Extract Regulates Expression of Genes Associated with Oxidative Stress and Inflammation in Human Endothelial Cells
Source: Molecules. 2019 Sep 12;24(18):3321. doi: 10.3390/molecules24183321 (PMC6767043; doi:10.3390/molecules24183321)
Supplement: Supplementary file 1 [file molecules-24-03321-s001.pdf]

## Supplementary Materials

Key findings from Rao, S., A.B. Santhakumar, K.A. Chinkwo, G. Wu, S.K. Johnson, and C.L. Blanchard, J. Cereal Sci., 2018, 84, 103; are listed below.

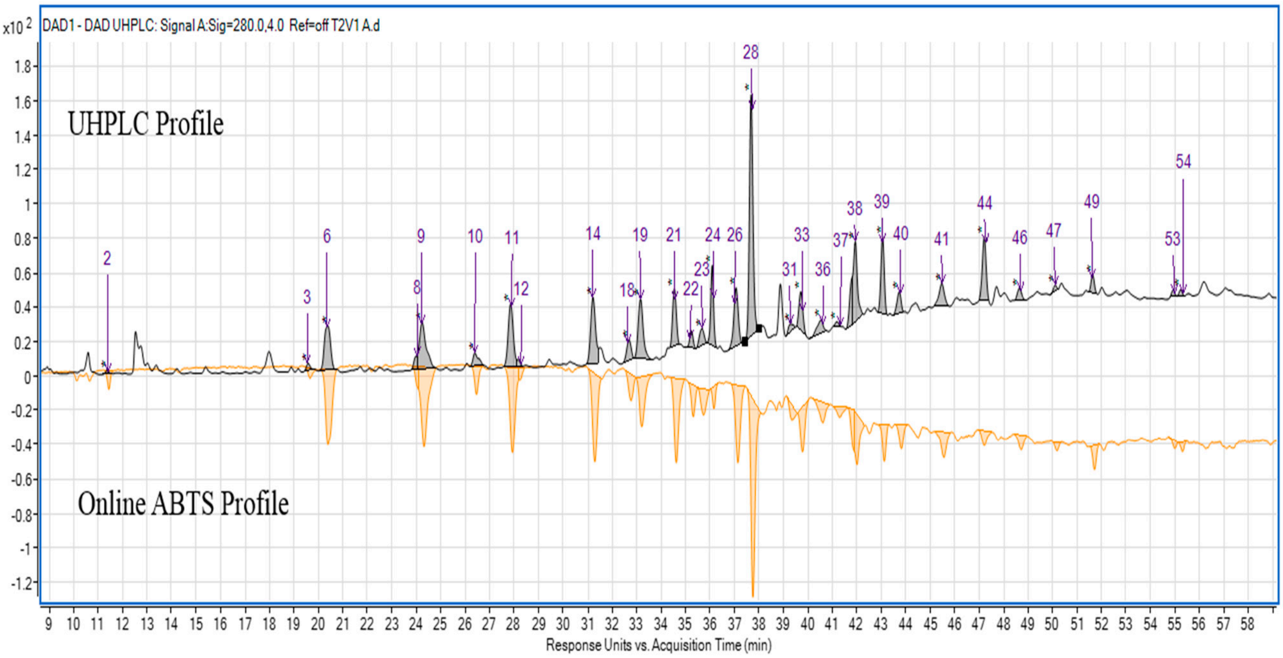


**Figure 1**. UHPLC online ABTS mapping of phenolic compounds from black pericarp sorghum variety Shawaya short black 1.

**Table 1.** Phenolic composition and antioxidant activity of sorghum varieties on as is basis.

| Variety | TPC mg/g GAE | TPAC mg/g CE | DPPH mg/g TE | FRAP mg/g TE |
|---|---|---|---|---|
| Shawaya short black 1 | 11.50 ± 1.81$_a$ | 3.02 ± 0.72$_a$ | 18.04 ± 3.53$_a$ | 20.92 ± 2.69$_a$ |
| IS 13116 (Brown) | 3.58 ± 1.63$_b$ | 5.55 ± 0.40$_b$ | 21.02 ± 5.17$_a$ | 4.62 ± 3.19$_b$ |
| QL33/QL36 (red) | 0.88 ± 0.17$_c$ | 0.47 ± 0.23$_c$ | 1.17 ± 0.48$_b$ | 4.83 ± 2.54$_b$ |
| B923296 (red) | 0.66 ± 0.27$_c$ | 0.41 ± 0.28$_c$ | 0.41 ± 0.14$_b$ | 2.72 ± 1.35$_b$ |
| QL12 (white) | 0.24 ± 0.03$_c$ | 0.09 ± 0.26$_c$ | 0.33 ± 0.10$_b$ | 2.31 ± 1.55$_b$ |
| QL33 (red) | 0.75 ± 0.23$_c$ | 0.60 ± 0.39$_c$ | 0.76 ± 0.18$_b$ | 3.43 ± 2.37$_b$ |

Different letters in the different columns represent significant difference at $p<0.05$. CE (Catechin equivalents), DPPH (2, 2-diphenyl-1-picrylhydrazyl), FRAP (Ferric reducing ability of plasma assay), GAE (Gallic acid equivalents), TE (Trolox equivalents), TPC (Total phenolic content) and TPAC (Total proanthocyanidin content).

**Table 2**. List of top ten phenolic compounds identified in the black sorghum phenolic rich extracts by Q-TOF LC/MS and quantified using UHPLC-Online ABTS system (Adapted from Rao et al., 2018)

| **Compound** | **Phenolic quantity (mg 100mg$^{-1}$ GAE)** | **Antioxidant activity (mg 100mg$^{-1}$ TE)** |
|---|---|---|
| Catechin derivative | 2.11 ± 0.47 | 1.51 ± 0.27 |
| Catechin | 1.97 ± 0.36 | 1.54 ± 0.25 |
| Pentahydroxyflavanone-(3->4)-catechin-7-O-glucoside | 1.80 ± 0.28 | 2.62 ± 0.36 |
| Catechin derivative | 0.66 ± 0.13 | 1.42 ± 0.24 |
| Pyrano-eriodictyol-(3->4)-catechin-7-O-glucoside | 0.65 ± 0.14 | 0.78 ± 0.16 |
| 1-O-Caffeoylglycerol-O-glucoside | 0.65 ± 0.12 | 1.74 ± 0.33 |
| N′.n′-dicafferoylspermidine | 0.61 ± 0.12 | 0.98 ± 0.15 |
| Pyrano-eriodictyol-(3->4)-catechin-7-*O*-glucoside isomer | 0.55 ± 0.08 | 0.60 ± 0.06 |
| Robinetinidol-(4alpha->6)-catechin-(6->4alpha)-robinetinidol | 0.53 ± 0.11 | 1.25 ± 0.20 |
| Pyrano-naringenin-(3->4)-catechin-7- *O*-glucoside isomer | 0.53 ± 0.07 | 0.42 ± 0.06 |

**Table 3.** Amplification efficiency of primers used in this study determined after running a standard curve.

| **Gene** | **Amplification efficiency - E (%)** |
|---|---|
| Nrf2 | 107% |
| NQO1 | 101.6% |
| HO1 | 101.5% |
| NOX4 | 107% |
| eNOS | 94.1% |
| MCP1 | 95.2% |
| ICAM1 | 100.1% |
| CD39 | 101% |
| CD73 | 93% |
| β-Actin | 95.5% |
